# Core neurological examination items for neurology clerks: A modified Delphi study with a grass-roots approach

**DOI:** 10.1371/journal.pone.0197463

**Published:** 2018-05-17

**Authors:** Chi-Hung Liu, Li-Ling Hsu, Cheng-Ting Hsiao, Suh-Ing Hsieh, Chun-Wei Chang, Elaine Shinwei Huang, Yeu-Jhy Chang

**Affiliations:** 1 Department of Neurology, Chang Gung Memorial Hospital, Linkou Medical Center, Taoyuan, Taiwan; 2 College of Medicine, Chang Gung University, Taoyuan, Taiwan; 3 Division of Medical Education, Graduate Institute of Clinical Medical Sciences, College of Medicine, Chang Gung University, Taoyuan, Taiwan; 4 Department of Nursing, Oriental Institute of Technology, New Taipei, Taiwan; 5 Department of Emergency Medicine, Chang Gung Memorial Hospital, Chiayi, Taiwan, School of Traditional Chinese Medicine, Chang Gung University, Taoyuan, Taiwan; 6 Department of Nursing, Chang Gung University of Science and Technology and Department of Nursing, Taoyuan Chang Gung Memorial Hospital, Taoyuan, Taiwan; 7 Chang Gung Medical Education Research Centre, Taoyuan, Taiwan; Taipei Veterans General Hospital, TAIWAN

## Abstract

**Background:**

With the evolution of treatments for neurological diseases, the contents of core neurological examinations (NEs) for medical students may need to be modified. We aimed to establish a consensus on the core NE items for neurology clerks and compare viewpoints between different groups of panelists.

**Methods:**

First, a pilot group proposed the core contents of NEs for neurology clerks. The proposed core NE items were then subject to a modified web-based Delphi process using the online software “SurveyMonkey”. A total of 30 panelists from different backgrounds (tutors or learners, neurologists or non-neurologists, community hospitals or medical centers, and different academic positions) participated in the modified Delphi process. Each panelist was asked to agree or disagree on the inclusion of each item using a 9-point Likert scale and was encouraged to provide feedback. We also compared viewpoints between different groups of panelists using the Mann-Whitney U test.

**Results:**

Eighty-three items were used for the first round of the Delphi process. Of them, 18 without consensus of being a core NE item for the neurology clerks in the first round and another 14 items suggested by the panelists were further discussed in the second round. Finally, 75 items with different grades were included in the recommended NE items for neurology clerks.

**Conclusions:**

Our findings provide a reference regarding the core NE items for milestone development for neurology clerkships. We hope that prioritizing the NE items in this order can help medical students to learn NE more efficiently.

## Introduction

Neurology is regarded as a difficult component of the medical curriculum. Neurophobia is defined as a fear of neurological diseases, and it is a recognized problem among medical students which may prevent them using their basic neurological knowledge at the bedside [1–6]. In addition, this can impede a young doctor’s motivation and confidence of learning neurology and being a neurologist [2, 4, 7, 8]. Neurophobia may start early in medical school [9]. Previous studies have suggested effective strategies to cure neurophobia [9]. Of them, transformation of teaching methods in neurological examinations (NEs) has been shown to play an important role [9]. Recently, the concept of milestone development has been integrated into medical education [10]. Different levels of learners should have different entrustable professional activities (EPAs) and corresponding skills in NEs [11–13]. Well trained doctors should be able to efficiently perform task-specific NEs to target the chief complaints and main symptoms, however, performing comprehensive NEs is difficult for beginners. Inadequate preparedness has been associated with stress and anxiety in medical students [14]. It is reasonable that students should learn about NEs sequentially, starting with the basic but essential items and then advancing to comprehensive knowledge. The essential NE items have previously been established in the core curriculum of neurology clerkship [15]. With the evolution in epidemiological distribution and treatment of neurological diseases, investigations are needed to determine whether the contents of NE education should be modified in the current era.

Using a modified Delphi process [16–18], the aim of this study was to update the consensus on core NE items that should be taught during the NE milestones in neurology clerkships based on the concept of a grass-roots approach [19, 20]. Consensus was made by panelists with different backgrounds, and in particular learners and clinical tutors. We also compared viewpoints between different groups of panelists.

## Materials and methods

### Definition of neurology clerkship

In Taiwan, medical students enter medical school after graduating from senior high school and study there for 6 years. Thereafter, they need to receive 2 years of post-graduate-year training, after which they can apply for residency training. Neurology clerks are 5^th^ grade medical students who have completed basic medical subjects and lectures on clinical neurology before starting clinical rotation. These students have very little experience in clinical neurology or performing NEs. We assumed that these students would be more likely to experience frustration when encountering the complicated and numerous items of comprehensive NEs.

### The pilot group

**Fig 1:**
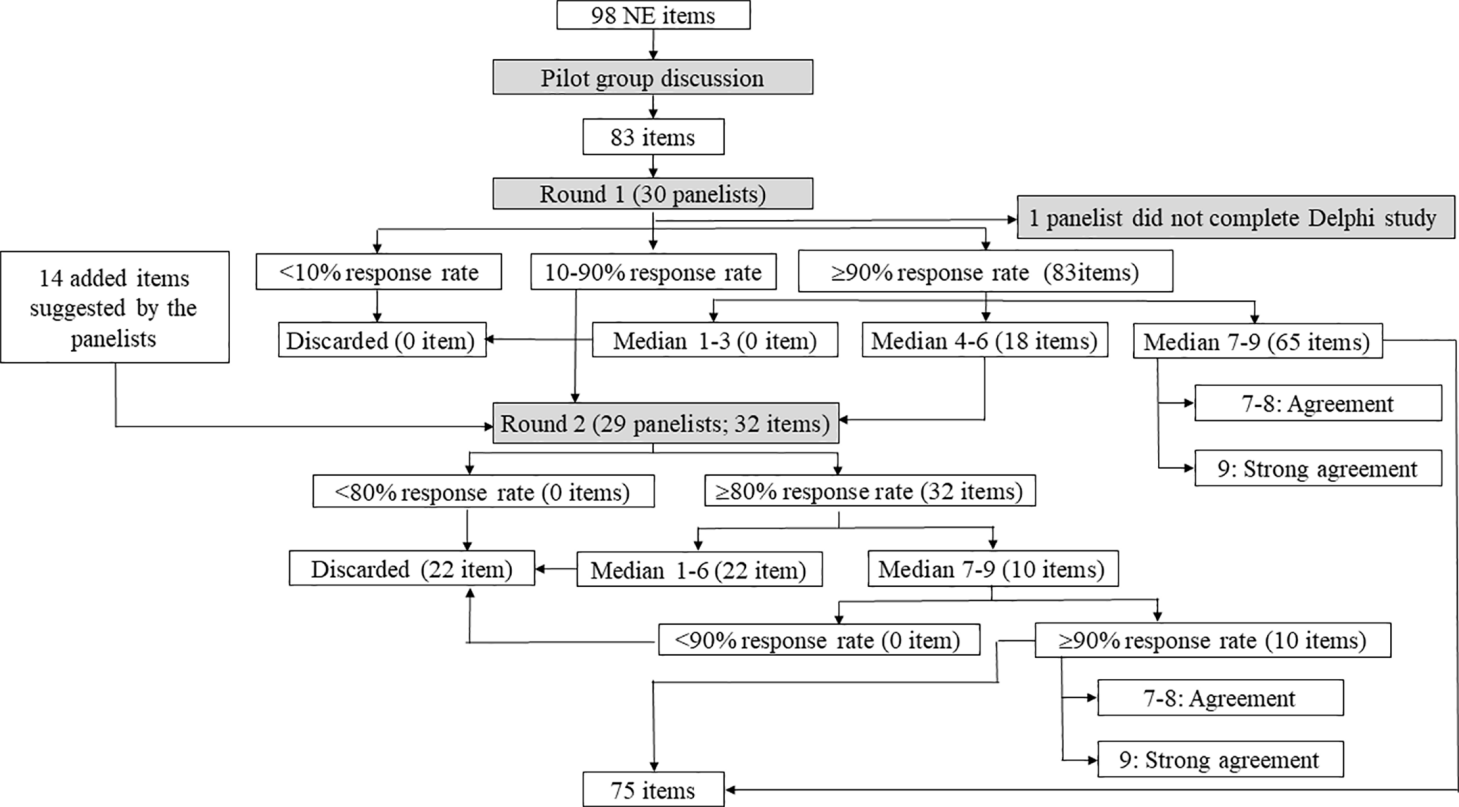
Flow charts of the pilot group and modified Delphi process. This figure showing the evolution and decision making process of all of the NE items concluded from the pilot group and each round of the modified Delphi process. NE = neurological examinations.

We enrolled five attending physicians from the Neurology Department of Chang Gung Memorial Hospital in the pilot group (Fig 1). These participants had different subspecialties in neurology and had actively contributed to medical education for more than 3 years. The objective of the pilot group was to generate a list of proposed core NE items for neurology clerks using NE textbooks (e.g. DeJong’s The Neurologic Examination, 7^th^ edition) as a reference using a LINE (an online chat application) discussion group. These items were then used in a Delphi process (S1 and S2 Tables).

### The modified Delphi process

#### The panelists

Representativeness and diversity are key factors for panelist selection in the modified Delphi process, and feedback on the elements of training may help to improve this problem [21, 22]. The needs of learners and clinical tutors are important in a grass-roots approach [19]. Therefore, professors, clinical tutors, and learners were the main targets for the panelists in this study [23]. A total of 30 panelists were enrolled for the modified Delphi process, who were selected from different learning (tutors and learners), expertise (neurologists and non-neurologists), levels of teaching hospital (community hospitals and medical centers), and academic positions. All of the selected learner panelists in this study had qualified academic performance in their neurology class (Table 1). As the neurology clerks may have had limited experience in neurology and therefore may have underestimated or overestimated the true significance of each NE item, we selected the learner panelists from different levels (medical students, interns, and residents). Six general neurologists came from community hospitals. We also enrolled 12 experts from medical centers with different subspecialties, including six from Chang-Gung Memorial Hospital and six from other medical centers. The Ethics Institutional Review Board of Chang Gung Memorial Hospital approved this study (104-9585B), and all of the panelists provided written inform consent.

**Table 1:** Selection criteria of the enrolled panelists.

Panelists	Selection criteria
Clerks	Academic performance within the top 50% in their same degree
Intern doctors	Academic performance within the top 50% in their same degree
Residents	Clinical performance within the top 50% in their same degree
Experts	Have >5 years of both educational and clinical experience as an attending physician of the neurological department in medical centers
General neurologists	Have >3 years of clinical experience as a general neurologist in community hospitals

#### The survey tool

In the traditional Delphi process, meetings should be held with all of the participants. This is time consuming, expensive, and difficult to arrange the attendance of all the clinical physicians. Therefore, in this study, we designed a web-based online anonymous modified Delphi process. We used the online software SurveyMonkey (SM; https://zh.surveymonkey.com/) to overcome the problems of distance and time. Personal e-mails were used as the main connection between the principal investigator and the panelists. SM sent e-mails to each panel member with a URL linking to the survey. We first sent a greeting e-mail with educational background surveys before the first round of the modified Delphi process. This step helped to reveal the self-reported expertise, age, and years of neurological education of the panelists. We also sent follow-up reminder e-mails to the non-responders in each round. Each survey round was available for 1 week. We copied the list of responders from each round into new recipient lists for subsequent rounds.

#### The first round

In the first round, we requested the panelists to evaluate whether an item should be included or excluded from the core NE items for the neurology clerks. We asked each panelist to agree or disagree with the inclusion of each item using a 9-point Likert scale, from 1 (strongly disagree) to 9 (strongly agree). We discarded the items if less than 10% of the panelists responded to them. We then discussed the items with response rates between 10% and 90% in the next round, and discussed the items with a response rate over 90% in this round. Of them, we defined the items with a score of 9 as strong agreement, and those with scores of 7 and 8 as agreement. The items with the scores between 4 and 6 were also discussed in the next round. We discarded the items with scores between 1 and 3. We asked the 30 panelists to provide feedback, and we also asked them to suggest NE items that were not initially included. We also confirmed the opinions of the panelists by phone to make sure that their suggestions were expressed correctly. The suggested NE items were further evaluated in the next round (Fig 1 and S1 Table).

#### The second round

We revised the proposed core NE items according to the suggestions and ratings in the first round, and drafted a revised list for the second round. In the second round, we discarded the items if less than 80% of the panelists responded to them, and discussed the items with a response rate of over 80%. Of these items, we discarded the items with scores between 1 and 6. For the items with scores between 7 and 9, those with a response rate less than 90% were also discarded. Following this round, all items were either included or excluded and consensus was achieved (Fig 1 and S1 Table).

### Data collection

We recorded the percentage of agreement of the NE items and used the 9-point Likert scale to register the importance of these items in each round of the modified Delphi process. Finally, we generated a recommended core list of NE items for neurology clerks.

### Statistical analysis

All statistical analyses were performed using the Statistical Package for the Social Sciences (IBM SPSS Statistics version 22.0). The Likert scale rating for each NE item from all of the panelists was expressed as median (quartile 1, quartile 3). Items with median scores between 7 and 9 in the first or second round were regarded to be recommended NE items. In addition, we further grouped the panelists based on their backgrounds (tutors and learners, neurologists and non-neurologists, experts in medical center and others) to compare differences in perspective between them using the Mann-Whitney U test (nonparametric data). Statistical significance was set at p < 0.05.

## Results

### The proposed core NE items for neurology clerks from the pilot group

In total, 98 items were initially provided for open discussion in the pilot group meeting (S2 Table). Some of these items were discarded as they were considered to be too difficult for medical students or combined to make the topic more comprehensive. Finally, 83 items were included in the proposed core list of NE items for further discussion in the modified Delphi process (Fig 1 and S3 Table).

### Background characteristics of the panelists

Among the panelists, 12 (40%) had experience of performing NEs for 0–10 years, and 18 (60%) had more than 11 years of experience. Fourteen of the panelists (46.7%) had experience of instructing NEs to students for more than 11 years, and 8 (26.7%) had less than 10 years of experience. The panelists included 12 learners (40%) and 18 tutors (60%). Among the 18 tutors, three (16.7%) were professors, four (22.2%) were associate professors, and five (27.8%) were assistant professors. Overall, 33.3% of the tutors were general neurologists in community hospitals. In addition, 10 (55.6%) of the 18 tutors had experience in curriculum design or had served as a program director. The most common expertise of the panelists was general neurology (55.6%), followed by headache (38.9%), cerebrovascular disease (33.3%), and neuro-critical care (33.3%). The panelists in this study covered most fields of subspecialties of clinical neuroscience (Table 2).

**Table 2:** Background characteristics of the panelists.

Variables			n (%)
Time from first performing a NE on patients			
	0–10 years		12(40.0)
	11–20 years		12(40.0)
	>20 years		6(20.0)
Time from first time instructing a NE to students			
	Never		8(26.7)
	0–10 years		8(26.7)
	11–20 years		8(26.7)
	>20 years		6(20.0)
Position			
	Learners 12 (40%)		
		Year 4 medical students	2(6.7)
		Clerks	2(6.7)
		Intern doctors	2(6.7)
		Post-graduate year 1 residents	2(6.7)
		Residents (non-neurology)	2(6.7)
		Residents (neurology)	2(6.7)
	Tutors 18 (60%)		
		General neurologists	6(20.0)
		Experts from CGMH	6(20.0)
		Experts from other medical centers	6(20.0)
Subspecialties and academic rank of the tutors			
	Academic rank		
		Professor	3(16.7)
		Associate professor	4(22.2)
		Assistant professor	5(27.8)
		General neurologists	6(33.3)
	Subspecialties		
		General neurology	10(55.6)
		Cerebrovascular disease	6(33.3)
		Cognitive neuroscience and dementia	3(16.7)
		Epilepsy	3(16.7)
		Movement disorder	3(16.7)
		Headache	7(38.9)
		Neuromuscular disease	5(27.8)
		Neurological infection	1(5.6)
		Neuro-critical care	6(33.3)
		Genetics in neurology	1(5.6)
		Neurorehabilitation	1(5.6)
		Sleep medicine	2(11.1)
		Clinical neurophysiology	3(16.7)
		Medical education	2(11.1)

CGMH = Chang Gung Memorial Hospital, NE = neurological examination.

### Results of the first round of the modified Delphi process

Twenty-nine (96.7%) of the 30 panelists completed the ratings. All 83 of the core proposed NE items were used for the first round of the modified Delphi process, of which 65 (78.3%) had agreement or strong agreement. Of these 65 items, 16 were strongly recommended as the core competence of neurology clerkship with a rating of 9 (S4 Table). However, 18 (21.7%) of the 83 items were put in the second round for further confirmation. Another 14 items were suggested to be added to the core list of NE items by the panelists in the first round (Fig 1). The panelists mentioned the main reasons why these items were considered to be important for neurology clerks in the “comment column” of the online questionnaires (S5 Table).

### Results of the second round of the modified Delphi process

The 18 items that were not considered to be core competence for neurology clerks in the first round were discarded after thorough reconsideration in the second round of the modified Delphi process. The ratings of these items were consistent between the two rounds. Ten of the 14 (71.4%) added items suggested by the panelists were regarded to be core competence for neurology clerkship (S5 Table). The 22 items discarded during the Delphi process were summarized in the S6 Table. Finally, 75 items were included in the recommended core list of NE items for neurology clerks (Fig 1 and Table 3).

**Table 3:** Results of the modified Delphi process.

Categories		Rating scores	
	9 (Strong agreement)	8 (Agreement)	7 (Agreement)
Physical examinations		1. Check pulse and heart rate	1. Listen to the heart sounds
		2. Check breathing sounds	2. Check thyroid goiters
			3. Check neck lymph node examination
			4. Listen to carotid bruit
			5. Check Kayser-Fleischer rings
Conscious and cognitive functions	1. Glasgow coma scale	1. Check complete Mini-Mental State Examination	1. Check language function (reading, writing, repetition, comprehension, fluency, naming)
		2. Understanding the definition of coma, semi-coma, stupor, confusion, delirium, and dementia	2. Check hemi-neglect
			3. Clock drawing test
Cranial nerves	1. Check pupil size and shape	1. Check visual field by confrontation test	1. Check visual acuity by eye chart
	2. Check direct light reflex	2. Check indirect light reflex and relative afferent pupillary defect	2. Check accommodation reflex
	3. Check eye movements	3. Check upper eye lid for ptosis	3. Check eye saccadic or pursuit movement
	4. Check facial sensations	4. Check nystagmus	4. Check eye convergent or divergent movement
	5. Check facial nerve function	5. Clenched teeth	5. Check onion skin sensation
		6. Check cornea reflex	6. Check jaw jerk
		7. Check hearing by finger rub screening test	7. Check Weber-Rinne test
		8. Check vestibulo-ocular reflex	8. Check uvula movement
		9. Gag reflex	
		10. Check shrugging shoulders or head turning to each side against hand	
		11. Check tongue movement	
Motor system	1. Check the distal and proximal muscle strength (MRC grading)	1. Check the muscle strength of different myotomes	1. Check the muscle strength of different nerves
		2. Check pronator drift	2. Check muscle bulk and volume
			3. Check Gower sign
			4. Could observe fasciculation
Sensation		1. Check light touch at arms/hands and legs/feet on both sides	1. Check temperature sensations, and compare the sensations between left/right side and proximal/distal side
		2. Check pinprick sensations, and compare the sensations between left/right side and proximal/distal side	2. Check the truncal sensation of different dermatomes
		3. Check vibration sensations and compare the sensations between left/right side and proximal/distal side	3. Check cortical sensation
		4. Check joint position sensation	
Reflexes	1. Check biceps, triceps, brachioradialis, patellar, and Achilles reflexes	1. Check Hoffmann' reflex	1. Perform methods of reinforcing the patellar reflex
	2. Check Babinski sign		2. Check clonus
Cerebellum	1. Check finger nose finger test		1. Check muscle tone
	2. Check heel-knee-shin test		2. Check scanning speech
	3. Check rapid alternative movement test		
Extrapyramidal systems		1. Check rigidity or spasticity in upper/lower limbs and neck	1. Check bradykinesia by finger tapping movement
			2. Describe the phenomenology of abnormal movements, including dystonia, spasticity, rigidity, tremor, chorea, ballism, and athetosis
Gait and stance	1. Check tandem gait	1. Observe the gait (arm swing, walk on heels, walk on toes, and turn en bloc)	
	2. Check Romberg test	2. Understanding abnormal gait, including hemiplegic gait, dystonic gait, scissors gait, wide base gait, festinating gait, gait apraxia	
Autonomic system	1. Ask about urine or stool incontinence	1. Check supine/standing blood pressure and heart rate	
		2. Understanding the Horner syndrome	
Others	1. Check meningeal irritation (Brudzinski's sign and Kernig's sign)	1. Straight leg raising test	1. Check National Institute of Health Stroke Scale
			2. Assess basic mood condition

MRC = Medical Research Council

### Differences in viewpoints between the panelists with diverse backgrounds

#### Tutors vs. learners

Twelve learners (residents or students) and 17 tutors (attending physicians) completed the questionnaires. Between these two groups, the main differences in viewpoint were touching the pharyngeal wall with a cotton wool stick (tutors vs. learners, 7 vs. 9, p = 0.04), checking muscle strength innervated by different nerves (tutors vs. learners, 6 vs. 8, p = 0.03), checking breathing sound (tutors vs. learners, 7 vs. 9, p = 0.01), neck lymph node examination (tutors vs. learners, 6 vs. 9, p < 0.01), asking about erectile function (tutors vs. learners, 5 vs. 6, p = 0.04), starch test (tutors vs. learners, 2 vs. 3.5, p = 0.02), and assessing basic mood status (tutors vs. learners, 7 vs. 8.5, p = 0.02) (Table 4).

**Table 4:** Differences in the viewpoints between the panelists with diverse backgrounds.

	Overall	Tutors vs. learners			Neurologists vs. non-neurologists			Experts in medical center vs. others		
Items of NE		Tutors	Learners	p	Neurologists	Non-neurologists	p	Medical center	Others	p
	n = 29*	n = 17	n = 12		n = 19	n = 10		n = 10	n = 19	
Check thyroid goiters	7(5, 7.5)	6(4, 7)	7(5.5, 8)	.130	6(3, 7)	7(6.5, 8.3)	**.049**	5.5(5, 7)	7(4, 8)	.261
Listened to carotid bruits	7(6, 8)	7(6.5, 8.5)	6(3.8, 7)	.116	7(7, 9)	6(2.8, 7)	**.025**	7(4.5, 8.3)	7(6, 8)	.795
Check upper eye lid for ptosis	8(7, 9)	8(7.5, 9)	7.5(7, 8)	.117	8(7, 9)	7.5(6.8, 8)	.081	8.5(6.8, 9)	8(7, 9)	.431
Check eye movements	9(9, 9)	9(9, 9)	9(8, 9)	.209	9(9, 9)	9(8, 9)	.090	9(9, 9)	9(8, 9)	.297
Check vertical gaze	6(5, 9)	7(5, 9)	6(4.3, 7.5)	.190	7(5, 9)	5.5(3.8, 6.5)	.072	6.5(3.5, 9)	6(5, 9)	.833
Check lacrimation / salivation	5(3, 7)	5(2.5, 6)	5.5(3.5, 7.8)	.107	4(3, 6)	6.5(5, 8)	**.016**	4.5(1.8, 6.3)	5(3, 7)	.341
Check caloric test	5(3, 6)	4(1.5, 5.5)	5(4, 6)	.122	4(2, 5)	5.5(3.8, 6.3)	.099	4.5(2, 5.3)	5(3, 6)	.500
Gag reflex	8(6, 9)	7(4.5, 9)	9(7.3, 9)	**.036**	8(5, 9)	9(7, 9)	.162	7(5.8, 8.3)	9(7, 9)	.135
Check the muscle strength of different nerves	7(5.5, 9)	6(5, 8.5)	8(7, 9)	**.026**	6(5, 9)	8(6.8, 9)	.064	6(4.5, 9)	8(6, 9)	.301
Check pronator drift	8(6.5, 9)	8(8, 9)	7.5(6, 8.8)	.151	9(8, 9)	7(5.8, 8)	**.019**	8.5(7.5, 9)	8(6, 9)	.337
Check Gower sign	7(6.5, 9)	8(6.5, 9)	7(5.5, 8)	.116	8(7, 9)	7(4.5, 8)	.053	8.5(6.8, 9)	7(5, 8)	.103
Check muscle tones	7(5.5, 9)	7(5, 8.5)	8(6, 9)	.364	7(5, 8)	8.5(6.8, 9)	.091	8(5, 9)	7(6, 9)	.724
Check rigidity or spasticity in upper/lower limbs and neck	8(5.5, 9)	8(7.5, 9)	6(5, 8.8)	.090	8(6, 9)	6.5(5, 9)	.318	8(6.5, 9)	8(5, 9)	.634
Check bradykinesia by finger tapping movement	7(5.5, 9)	9(6.5, 9)	6.5(5, 7.8)	.090	8(6, 9)	7(4.8, 8.3)	.296	7.5(5.3, 9)	7(5, 9)	.906
Check tandem gait	9(8, 9)	9(8.5, 9)	8.5(7.3, 9)	.229	9(9, 9)	8(6. 8,9)	.073	9(8.8, 9)	9(7, 9)	.193
Check breathing sounds	8(6, 9)	7(2.5, 9)	9(7.5, 9)	**.010**	7(3, 9)	9(8.5, 9)	**.015**	6(2, 7.3)	9(7, 9)	**.004**
Check pulse and heart rate	8(7, 9)	8(5.5, 9)	9(7.3, 9)	.257	8(6, 9)	9(7, 9)	.352	7.5(4.5, 8.3)	9(8, 9)	**.023**
Check neck lymph node examination	7(4, 9)	6(2.5, 7.5)	9(7, 9)	**.002**	6(3, 8)	9(7, 9)	**.005**	4(2, 7.3)	9(6, 9)	**.010**
Understanding the definition of coma, semi-coma, stupor, confusion, delirium, and dementia	8(8, 9)	8(7.5, 9)	9(8, 9)	.052	8(8, 9)	9(8, 9)	.209	8(6.5, 8.3)	9(8, 9)	**.016**
Ask about erection function	5(3, 7)	5(2.5, 5.5)	6(4.5, 8.5)	**.035**	5(3, 6)	6(3.8, 7.5)	.157	5(2.8, 6.3)	6(3, 7)	.430
Starch test	3(2, 4.5)	2(1, 3.5)	3.5(3, 6.5)	**.015**	2(1, 4)	3.5(2.8, 7)	**.040**	2.5(1, 3.3)	3(2, 5)	.155
Assess basic mood status	7(5.5, 9)	7(5, 8)	8.5(7, 9)	**.019**	7(5, 8)	8.5(6.8, 9)	**.044**	5.5(4.3, 7)	8(7, 9)	**.003**

Median (Q1, Q3) was used to report rating scores.

*One panelist did not complete the survey.

Data were analyzed using the Mann-Whitney U test. **Bold p** values are significant.

#### Neurologists vs. non-neurologists

Comparing the 19 neurologists (tutors and residents of the neurology department) with the 10 non-neurologists, the non-neurologists strongly favored checking lacrimation/salivation (neurologists vs. non-neurologists, 4 vs. 6.5, p = 0.02), breathing sounds (neurologists vs. non-neurologists, 7 vs. 9, p = 0.02), neck lymph node examination (neurologists vs. non-neurologists, 6 vs. 9, p < 0.01), starch test (neurologists vs. non-neurologists, 2 vs. 3.5, p = 0.04), and assessing basic mood status (neurologists vs. non-neurologists, 7 vs. 8.5, p = 0.05). The neurologists thought that listening to the carotid pulse (neurologists vs. non-neurologists, 7 vs. 6, p = 0.03), and checking pronator drift (neurologists vs. non-neurologists, 9 vs. 7, p = 0.02) were the most important NE items for neurology clerks (Table 4).

#### Experts in medical centers vs. others

The experts in medical centers considered that checking breathing sounds (experts in medical centers vs. others, 6 vs. 9, p < 0.01), checking pulse and heart rate (experts in medical centers vs. others, 7.5 vs. 9, p = 0.03), neck lymph node examination (experts in medical centers vs. others, 4 vs. 9, p < 0.01), understanding the definition of coma, semi-coma, stupor, confusion, delirium, and dementia (experts in medical centers vs. others, 8 vs. 9, p = 0.02), and assessing basic mood status (experts in medical centers vs. others, 5.5 vs. 8, p < 0.01) were less important NE items for neurology clerks (Table 4).

## Discussion

The 16 items with strong agreement in this study were identical to those in previous guidelines for a comprehensive NE for neurology clerks [15, 24]. Our study further highlights the importance of performing physical examinations in neurological patients. In addition, our findings emphasized the importance of NE items on the autonomic system, meningeal irritation, acute ischemic stroke (AIS) screening and patient selection for thrombolytic therapy, abnormal gait and phenomenology of abnormal movements. All of these factors can be associated with the advancements in diagnosis and treatment of neurological emergencies and the increased epidemiological trend of degenerative diseases.

Previous recommendations from the American Academy of Neurology (AAN) stated that dealing with neurological emergencies is essential content that should be taught to neurology clerks (https://www.aan.com/siteassets/home-page/tools-and-resources/academic-neurologist--researchers/clerkship-and-course-director-resources/neurology-clerkship-core-curriculum-guidelines.new.pdf). Meningitis and subarachnoid hemorrhage are common life-threatening neurological emergencies, and a delayed diagnosis of these diseases may lead to serious consequences. Checking meningeal irritation is a simple method to screen these diseases, however it was not listed in the guidelines for a comprehensive NE. Our panelists generally agreed that all neurology clerks should be familiar with this skill. With improvements in intravenous thrombolysis and mechanical thrombectomy, the aim of AIS treatment now includes the early detection and early reperfusion [25]. It is therefore reasonable that all primary physicians, not only neurologists, should be aware of the major signs of AIS and should have the ability to screen the patients who might be candidates for reperfusion therapy. In the current study, the tutors and neurologists placed more emphasis on the items of carotid bruit auscultation, pronator drift, and the National Institute of Health Stroke Scale. This suggests that AIS screening and stroke severity evaluation should be highlighted in the core curriculum for neurology clerks [26]. In addition, the epidemiological trend in the aging society raises the importance of degenerative diseases including dementia and movement disorders in medical education. Mental status and involuntary movements are listed in the AAN NE guidelines for neurology clerks. However, our results further highlight that neurology clerks should be able to holistically assess basic cognitive function using the Mini-Mental State Examination. We also identified the involuntary movements that neurology clerks should learn.

We ranked the importance of the core NE items using median scores. Far more recommended NE items were identified in the current study compared to the AAN NE guidelines. However, the contents were generally similar between the AAN NE guidelines and our items with median scores of 8 and 9 (Table 3). We further separated each NE item into several parts (e.g. we separated the light reflex examination into direct light reflex, indirect light reflex, and relative afferent pupillary defect) and rated them individually. Simple examinations to assess brain stem function of patients with stupor and coma should be a core EPA for all clinical doctors and not only neurologists. Evaluating muscle power, understanding the Medical Research Council grading system, checking sensations, deep tendon reflexes, Babinski sign, Hoffmann’ reflex, cerebellar signs, and Romberg test are also important. Therefore, understanding the anatomy of the motor, sensory, and cerebellar systems is crucial before learning these NE skills. Checking spasticity, rigidity, and bradykinesia, and understanding an abnormal gait were also emphasized in our findings. The items with a median score of 7 included common contents that were frequently taught to our learners. Our results indicate that these items, such as the phenomenology of abnormal movements, may still be important for the students who have become skilled at the NE items with median scores of 9 and 8. Interestingly, our learners did not give as high ratings to the items for examining movement disorders as the tutors and experts. However movement disorders are not uncommon in clinical practice. This suggests that it would be worthwhile to develop curriculum to improve students’ basic concepts of these presentations [27, 28]. We believe that prioritizing the items in this order can help neurology clerks to learn NE more efficiently.

Traditionally, panelists in the Delphi process are experts and program directors, and it is possible that learner panelists may not be as well experienced in performing NEs or in different clinical scenarios. This may potentially confound the results. However, the core NE items for neurology clerks should not be too difficult for beginners. But these items should still meet the learners’ needs for their future clinical practice. To fulfill this demand, a bottom-up grassroots approach could be a practical method [20, 29, 30]. User panelists can still be included in the Delphi process [31]. In the current study, we included the opinions from learners and general neurologists. To reduce this bias, we assured the dominance of the tutors (60%) in the composition of the panelists, and we carefully selected the learners from different levels. The learners’ academic performance was within the top 50% in their same degree. We believe that this could reduce any confounding effect. Moreover, we further compared the viewpoints between the learners and tutors. This demonstrated a consensus with the tutors’ opinions only (S7 Table). The results showed small differences in the viewpoints between the learners and tutors, which is similar to previous studies [24]. However our learners and non-neurologists tended to give higher ratings to the NE items than the tutors, neurologists, and experts. It is possible that the learners and non-neurologists did not have enough clinical experience of patients with neurological diseases, and therefore they may have been more anxious of missing an important element of a NE that may lead to an incorrect diagnosis [14]. In addition, the learners and non-neurologists may have been less familiar with the neuroanatomy and clinical features of neurological diseases, and thus they felt the need to perform comprehensive NEs to reduce errors. In contrast, the neurologists tended to integrate the NE items instead of analyzing them separately, and thus they could better perform a hypothesis-driven rather than screening NE [32]. Based on these results, we suggest that medical students should focus on simplified core NE items before they have experience of neurological diseases and clinical care. In addition, it may be more important to establish a core NE structure than to comprehensively introduce a wide range of NE items for neurology clerks. It is essential to design an integrated curriculum which incorporates NE skills with regards to knowledge and clinical practice for neurological diseases, and this may facilitate medical students to use cognitive and meta-cognitive strategies [33]. The ability to integrate hypothesis-driven NEs into their practice may also help to improve motivation and self-regulated learning [34].

The strengths of this study include the web-based modified Delphi study, which could overcome the barriers of distance, time, and expense [35]. In addition, the heterogeneity of the members may have resulted in a better performance. We tried to expand the heterogeneity of the panelists, and we believe that this may have improved the results [31]. Adhesion is crucial to the results of the Delphi process, and we used the following methods to improve adhesion. First, we sent a letter explaining the methods to all of the panelists before the beginning of the study to help them understand the sequence of the study. Second, we used an online survey tool instead of e-mails to accomplish this study. The panelists could perform ratings on multiple devices including a computer, tablet, or smartphone. The ratings could thus be completed anywhere and at any time if their device could connect to the internet. The convenience of the online tool led to the high adhesion rate (96.7%) in this study. However, there are still several limitations to this study. First, the panelists who were not familiar with mobile devices may have had difficulty in completing the questionnaires. However, we supplied printed versions to these panelists if they complained about the inconvenience of using the online tools. Second, online panel discussions also have potential disadvantages including lower levels of interaction and engagement [36]. The web-based Delphi process may have limited free discussion among the panelists during the brainstorming process, and this may have particularly influenced the consensus gathering process with regards to the disputed items. To reduce this bias, we recorded the panelists’ opinions and provided their comments to all of the panelists separately by phone if requested. Finally, not all of the panelists were course directors, and we did not enroll international panelists. These factors may have influenced the generalizability of our findings to other countries.

## Conclusions

Through our modified Delphi process, we provided a reference regarding the core NE items corresponding to the milestones of neurology clerkship. We hope our results can help to integrate clinical neurology and NE skills into a curriculum design and focus the content on the core issues. Future studies are needed to develop an assessment tool for NEs and to evaluate the reliability and validity of that tool.

## Supporting information

S1 Table Delphi: Flow chart. (DOCX)

S2 Table Items of neurological examinations initially provided for the pilot group. (DOCX)

S3 Table List of proposed core neurological examination items for the modified Delphi process. (DOCX)

S4 Table Results of the first round modified Delphi process. (DOCX)

S5 Table Results of the second round modified Delphi process. (DOCX)

S6 Table Summary of the 22 items discarded during the Delphi process. (DOCX)

S7 Table Results of the modified Delphi process from the tutors’ opinions. (DOCX)
