# Supplementary material for: Core neurological examination items for neurology clerks: A modified Delphi study with a grass-roots approach
Source: PLoS One. 2018 May 17;13(5):e0197463. doi: 10.1371/journal.pone.0197463 (PMC5957356; doi:10.1371/journal.pone.0197463)
Supplement: S1 Table — (DOCX) [file pone.0197463.s001.docx]

| S1 Table. Delphi: Flow chart | | |
| --- | --- | --- |
| Process |  | Details of each process |
| Pilot group |  | 1. 5 participants were enrolled. |
|  |  | 2. The participants were asked to draft proposed core NE items for the neurology clerks. |
|  |  | 3. 83 items of NE were included in the proposed core NE items, which were provided for the modified Delphi discussion. |
| Modified Delphi round 1 |  | 1. 30 panelists were included in the modified Delphi process. |
|  |  | 2. The panelists were asked to agree or disagree on every item of the proposed core NE using a 9-point rating scale. |
|  |  | 3. The items would be discussed in this round if over 90% of participants responded to survey. |
|  |  | 3.1 Items with median score “9” were registered as strong agreement. |
|  |  | 3.2 Items with median scores “7 and 8” were registered as agreement. |
|  |  | 3.3 Items with median scores between “4 and 6” would be later discussed in the round 2. |
|  |  | 3.4 Items with median scores between “1 and 3” were discarded. |
|  |  | 4. The panelists were welcomed to provide feedback and add items that were not in the proposed core NE items. |
| Modified Delphi round 2 |  | 1. The same 30 panelists were included in the modified Delphi process. |
|  |  | 2. Totally 33 items of NE were discussed in the modified round 2, including: |
|  |  | 2.1 18 of the proposed core NE items with the median score between “4 and 6” in the round 1. |
|  |  | 2.2 15 items of NE those were added by the panelists in the round 1. |
|  |  | 3. The panelists were asked to agree or disagree on every item of in the round 2 using a 9-point rating scale. |
|  |  | 4. The items would be discussed in this round if over 80% of participants responded to survey. |
|  |  | 4.1 Items with median score “9” were registered as strong agreement. |
|  |  | 4.2 Items with median scores “7 and 8” were registered as agreement. |
|  |  | 4.3 Items with median scores between “1 and 6” were discarded. |
| Final of the modified Delphi |  | The recommended NE items for the neurology clerks were achieved |
| Note: NE, neurological examination | | |
