# Supplementary material for: Core neurological examination items for neurology clerks: A modified Delphi study with a grass-roots approach
Source: PLoS One. 2018 May 17;13(5):e0197463. doi: 10.1371/journal.pone.0197463 (PMC5957356; doi:10.1371/journal.pone.0197463)
Supplement: S2 Table — (DOCX) [file pone.0197463.s002.docx]

S2 Table. Items of neurological examinations initially provided for the pilot group

|  |  |  |
| --- | --- | --- |
| Category | Item No | Checklist item |
| Physical examination | 1 | Listen to the heart sounds (PE) |
|  | 2 | Check the thyroid goiter (PE) |
|  | 3 | Listen to carotid bruits (PE) |
| High cortical function | 4 | Glasgow coma scale (GCS) |
|  | 5 | Check complete Mini-Mental State Examination (MMSE) (High cortical function) |
|  | 6 | Check complete Cognitive abilities screening instrument (CASI) (High cortical function) |
|  | 7 | Check complete Frontal assessment battery (FAB) (High cortical function) |
|  | 8 | Check complete Clinical Dementia Rating (CDR) (High cortical function) |
|  | 9 | Check language function (reading, writing, repetition, comprehension, fluency, naming) (High cortical function) |
|  | 10 | Check speech volume, pitch, rhythm (High cortical function) |
|  | 11 | Check glabellar sign and palmomental reflex (primitive reflex) (High cortical function) |
|  | 12 | Check calculation by seven series (High cortical function) |
|  | 13 | Check hemineglect by touch, finger rubbing, and finger moving on the both side simultaneously (High cortical function) |
|  | 14 | Check orientation to time, place, and person (High cortical function) |
|  | 15 | Check recent memory and immediately recalls (High cortical function) |
|  | 16 | Check agnosia (High cortical function) |
|  | 17 | Check apraxia (High cortical function) |
| Cranial nerves | 18 | Check smell by vinaigrette (I) |
|  | 19 | Check visual acuity by eye chart (II) |
|  | 20 | Check color vision (II) |
|  | 21 | Check visual field by confrontation test (II) |
|  | 22 | Check pupil size and shape (II) |
|  | 23 | Check direct light reflex (II, III) |
|  | 24 | Check indirect light reflex (II, III) and relative afferent pupillary defect (RAPD) |
|  | 25 | Check accommodation reflex (II, III) |
|  | 26 | Check eye fundus using fundoscope (II) |
|  | 27 | Check upper eye lid for ptosis |
|  | 28 | Check eye movements (III, IV, VI) |
|  | 29 | Check eye saccadic or pursuit movement (III, IV, VI) |
|  | 30 | Check eye convergent or divergent movement (III, IV, VI) |
|  | 31 | Check Bielschowsky head tilt test (IV) |
|  | 32 | Check vertical gaze (III, IV, VI) |
|  | 33 | Check nystagmus (III, IV, VI) |
|  | 34 | Check cover and uncover test (III, IV, VI) |
|  | 35 | Check optokinetic nystagmus |
|  | 36 | Using Maddox rod test |
|  | 37 | Clenched teeth (V motor) |
|  | 38 | Check facial sensation by cotton swab on forehead/cheeks/jaws while eyes closed (V sensation) |
|  | 39 | Check onion skin sensation (V sensation) |
|  | 40 | Check jaw jerk (V) |
|  | 41 | Check cornea reflex with cotton wool (V, VII) |
|  | 42 | Check facial nerve function by raising eyebrows/closing eyes tightly/smiling/ showing teeth (VII) |
|  | 43 | Check taste (VII) |
|  | 44 | Check lacrimation / salivation (VII) |
|  | 45 | Check hearing by finger rub screening test (VIII) |
|  | 46 | Check Weber /Rinne test by tuning fork (VIII) |
|  | 47 | Check vestibulo-ocular reflex (doll's eye test, head thrust) (VIII) |
|  | 48 | Check caloric test (VIII) |
|  | 49 | Check ‘ahh’ for uvula movement (IX, X) |
|  | 50 | Touch pharyngeal wall with cotton wool stick (Gag reflex) (IX, X) |
|  | 51 | Check shrugging shoulders while pressing down on them or check head turning to each side against hand (XI) |
|  | 52 | Check tongue movement (XII) |
| Motor system | 53 | Check the muscle strength distal and proximal on both sides (Motor function) |
|  | 53 | Check the muscle strength of different myotomes (Motor function) |
|  | 54 | Check the muscle strength of different nerves (Motor function) |
|  | 55 | Check muscle bulk and volume (Motor function) |
|  | 56 | Check pronator drift (Motor function) |
|  | 57 | Check Gower sign (Motor function) |
|  | 58 | Could observe fasciculation (Motor function) |
| Sensation | 59 | Check light touchat arms/hands and legs/feet on both sides (Sensation) |
|  | 60 | Check pinprick sensations, and compare the sensations between left/right side and proximal/distal side (Sensation) |
|  | 61 | Check temperature sensations, and compare the sensations between left/right side and proximal/distal side (Sensation) |
|  | 62 | Check vibration sensations using the tuning fork and compare the sensations between left/right side and proximal/distal side (Sensation) |
|  | 63 | Check joint position sensation (Sensation) |
|  | 64 | Check the truncal sensation of different dermatomes (Sensation) |
|  | 65 | Check cortical sensation (Sensation) |
| Reflexes | 66 | Check biceps reflex (Reflexes) |
|  | 67 | Check triceps reflex (Reflexes) |
|  | 68 | Check brachioradialis reflex (Reflexes) |
|  | 69 | Check patellar reflex (Reflexes) |
|  | 70 | Check Achilles reflex (Reflexes) |
|  | 71 | Perform method of reinforcing the patellar reflex (Reflexes) |
|  | 72 | Check finger flexor (Reflexes) |
|  | 73 | Check pectoralis reflex (Reflexes) |
|  | 74 | Check Babinski sign (Reflexes) |
|  | 75 | Check abdominal reflex (Reflexes) |
|  | 76 | Check Hoffmann' reflex (Reflexes) |
| Cerebellum | 77 | Check finger nose finger test (Cerebellum) |
|  | 78 | Check heel-knee-shin test (Cerebellumr) |
|  | 79 | Check rapid alternative movement test (Cerebellum) |
|  | 80 | Check muscle tones (Cerebellum) |
|  | 81 | Check scanning speech (Cerebellum) |
| Extrapyramidal systems | 82 | Check rigidity or spasticity in upper/lower limbs and my neck (Extrapyramidal system) |
|  | 83 | Check bradykinesia by finger tapping movement (Extrapyramidal system) |
|  | 84 | Check resting tremor by counting number when eye closed (Extrapyramidal system) |
|  | 85 | Check pull test (Extrapyramidal system) |
|  | 86 | Describe the phenomenology of abnormal movements (Extrapyramidal system) |
|  | 87 | Check UPDRS scale motor part (Extrapyramidal system) |
| Gait and station | 88 | Observe the gait (Gait) |
|  | 89 | Check walk on heels (Gait) |
|  | 90 | Check walk on toes (Gait) |
|  | 91 | Check tandem gait (Gait) |
|  | 92 | Check turn en bloc (Gait) |
|  | 93 | Check Romberg test (Gait) |
| Autonomic system | 94 | Ask about urine or stool incontinence (Autonomic nervous system) |
|  | 95 | Check supine/standing blood pressure and heart rate (Autonomic nervous system) |
| Others | 96 | Check Brudzinski's sign |
|  | 97 | Check Kernig's sign |
|  | 98 | Check National Institute of Health Stroke scale (NIHSS) |
