# Supplementary material for: Core neurological examination items for neurology clerks: A modified Delphi study with a grass-roots approach
Source: PLoS One. 2018 May 17;13(5):e0197463. doi: 10.1371/journal.pone.0197463 (PMC5957356; doi:10.1371/journal.pone.0197463)
Supplement: S3 Table — (DOCX) [file pone.0197463.s003.docx]

S3 Table. List of proposed core neurological examination items for the modified Delphi process.

| Categories |  | Items |
| --- | --- | --- |
| Physical examination |  | Listen to the heart sounds |
|  |  | Check thyroid goiters |
|  |  | Listen to carotid bruits |
| Conscious and cognitive functions |  | Glasgow coma scale |
|  |  | Check complete Mini-Mental State Examination |
|  |  | Check language function (reading, writing, repetition, comprehension, fluency, naming) |
|  |  | Check speech volume, pitch, rhythm |
|  |  | Check glabellar sign and palmomental reflexes |
|  |  | Check hemi-neglect by touch, finger rubbing, and finger moving on the both side simultaneously |
| Cranial nerves |  | Check smell by vinaigrette |
|  |  | Check visual acuity by eye chart |
|  |  | Check color vision |
|  |  | Check visual field by confrontation test |
|  |  | Check pupil size and shape |
|  |  | Check direct light reflex |
|  |  | Check indirect light reflex (II, III) and relative afferent pupillary defect |
|  |  | Check accommodation reflex |
|  |  | Check eye fundus using fundoscope |
|  |  | Check upper eye lid for ptosis |
|  |  | Check eye movements |
|  |  | Check eye saccadic or pursuit movement |
|  |  | Check eye convergent or divergent movement |
|  |  | Check Bielschowsky head tilt test |
|  |  | Check vertical gaze |
|  |  | Check nystagmus |
|  |  | Check cover and uncover test |
|  |  | Check optokinetic nystagmus |
|  |  | Clenched teeth |
|  |  | Check facial sensation by cotton swab on forehead/cheeks/jaws while eyes closed |
|  |  | Check onion skin sensation |
|  |  | Check jaw jerk |
|  |  | Check cornea reflex with cotton wool |
|  |  | Check facial nerve function by raising eyebrows/closing eyes tightly/smiling/ showing teeth |
|  |  | Check taste |
|  |  | Check lacrimation / salivation |
|  |  | Check hearing by Calibrated finger rub auditory screening test |
|  |  | Check Weber /Rinne test by tuning fork |
|  |  | Check vestibulo-ocular reflex (doll's eye test, head thrust) |
|  |  | Check Caloric test |
|  |  | Check Hallpike's test |
|  |  | Check “ahh” for uvula movement |
|  |  | Touch pharyngeal wall with cotton wool stick (Gag reflex) |
|  |  | Check shrugging shoulders while pressing down on them or check head turning to each side against hand |
|  |  | Check tongue movement |
| Motor system |  | Check the muscle strength distal and proximal on both sides |
|  |  | Check the muscle strength of different myotomes |
|  |  | Check the muscle strength of different nerves |
|  |  | Check muscle bulk and volume |
|  |  | Check pronator drift |
|  |  | Check Gower sign |
|  |  | Could observe fasciculation |
| Sensation |  | Check light touchat arms/hands and legs/feet on both sides |
|  |  | Check pinprick sensations, and compare the sensations between left/right side and proximal/distal side |
|  |  | Check temperature sensations, and compare the sensations between left/right side and proximal/distal side |
|  |  | Check vibration sensations using the tuning fork and compare the sensations between left/right side and proximal/distal side |
|  |  | Check joint position sensation |
|  |  | Check the truncal sensation of different dermatomes |
|  |  | Check cortical sensation |
| Reflexes |  | Check biceps, triceps, brachioradialis, patellar, and Achilles reflexes |
|  |  | Perform method of reinforcing the patellar reflex |
|  |  | Check finger flexor |
|  |  | Check pectoralis reflex |
|  |  | Check Babinski sign |
|  |  | Check Hoffmann' reflex |
|  |  | Check clonus |
| Cerebellum |  | Check finger nose finger test |
|  |  | Check heel-knee-shin test |
|  |  | Check rapid alternative movement test |
|  |  | Check muscle tones |
|  |  | Check scanning speech |
| Extrapyramidal systems |  | Check rigidity or spasticity in upper/lower limbs and my neck |
|  |  | Check bradykinesia by finger tapping movement |
|  |  | Check resting tremor by counting number when eye closed |
|  |  | Check pull test |
|  |  | Describe the phenomenology of abnormal movements, including dystonia, spasticity, rigidity, tremor, chorea, ballism, and athetosis |
|  |  | Check unified Parkinson's disease rating scale motor part |
| Gait and stance |  | Observe the gait (arm swing, walk on heels, walk on toes, and turn en bloc) |
|  |  | Check tandem gait |
|  |  | Check Romberg test |
| Autonomic system |  | Ask about urine or stool incontinence |
|  |  | Check supine/standing blood pressure and heart rate |
| Others |  | Check meningeal irritation (Brudzinski's sign and Kernig's sign) |
|  |  | Check National Institute of Health Stroke Scale |
