# Supplementary material for: Core neurological examination items for neurology clerks: A modified Delphi study with a grass-roots approach
Source: PLoS One. 2018 May 17;13(5):e0197463. doi: 10.1371/journal.pone.0197463 (PMC5957356; doi:10.1371/journal.pone.0197463)
Supplement: S4 Table — (DOCX) [file pone.0197463.s004.docx]

S4 Table. Results of the first round modified Delphi process

|  | Overalls |  | Tutors | Learners | p |  | Neurologist | Non-neurologists | p |  | Medical center | Others | p |
| --- | --- | --- | --- | --- | --- | --- | --- | --- | --- | --- | --- | --- | --- |
|  | (n = 29*) |  | (n = 17) | (n = 12) | p |  | (n = 19) | (n = 10) | p |  | (n = 10) | (n = 19) | p |
| Items of NE | Median(Q1, Q3) |  | Median(Q1, Q3) | Median(Q1, Q3) | value |  | Median(Q1, Q3) | Median(Q1, Q3) | value |  | Median(Q1, Q3) | Median(Q1, Q3) | value |
| Listen to the heart sounds | 7(5, 9) |  | 7(4.5, 8.5) | 7.5(5.3, 9) | .418 |  | 7(5, 8) | 8(5.8, 9) | .225 |  | 6.5(5, 8.3) | 7(5, 9) | .692 |
| Check thyroid goiters | 7(5, 7.5) |  | 6(4, 7) | 7(5.5, 8) | .130 |  | 6(3, 7) | 7(6.5, 8.3) | **.049** |  | 5.5(5, 7) | 7(4, 8) | .261 |
| Listen to carotid bruits | 7(6, 8) |  | 7(6.5, 8.5) | 6(3.8, 7) | .116 |  | 7(7, 9) | 6(2.8, 7) | **.025** |  | 7(4.5, 8.3) | 7(6, 8) | .795 |
| Glasgow coma scale | 9(9, 9) |  | 9(9, 9) | 9(8.3, 9) | .754 |  | 9(9, 9) | 9(8, 9) | .476 |  | 9(8.5, 9) | 9(9, 9) | .948 |
| Check complete Mini-Mental State Examination | 8(6, 9) |  | 8(5, 9) | 8(6.3, 8.8) | .586 |  | 8(5, 9) | 7.5(6, 8.3) | .963 |  | 7.5(4.8, 9) | 8(6, 9) | .622 |
| Check language function (reading, writing, repetition, comprehension, fluency, naming) | 7(6, 9) |  | 8(6, 9) | 6.5(5.3, 8) | .268 |  | 8(6, 9) | 6.5(5, 8) | .167 |  | 7.5(5, 8.3) | 7(6, 9) | .639 |
| Check speech volume, pitch, rhythm | 5(4, 7) |  | 5(4.5, 7) | 5(2.3, 6.8) | .309 |  | 5(4, 7) | 5(2, 6.3) | .292 |  | 5.5(3.8, 7) | 5(4, 7) | .708 |
| Check glabellar sign and palmomental reflexes | 6(4, 7.5) |  | 7(4.5, 7.5) | 5(4, 7.8) | .358 |  | 7(5, 7) | 4.5(3.8, 8) | .318 |  | 7(3.8, 8) | 6(4, 7) | .659 |
| Check hemi-neglect by touch, finger rubbing, and finger moving on the both side simultaneously | 7(6, 8) |  | 7(5.5, 8) | 7(6, 8) | .909 |  | 7(6, 8) | 7(5.8, 8) | .869 |  | 7.5(6. 5,8) | 7(6, 8) | .636 |
| Check smell by vinaigrette | 5(3, 6.5) |  | 3(3, 6) | 6(5, 7) | .185 |  | 5(3, 6) | 6(4, 7) | .245 |  | 4(3, 5.3) | 6(3, 7) | .352 |
| Check visual acuity by eye chart | 7(5.5, 8) |  | 6(5, 7.5) | 7(6, 8) | .184 |  | 6(5, 7) | 7.5(5.8, 8.3) | .148 |  | 6.5(5, 7.3) | 7(6, 8) | .608 |
| Check color vision | 6(3.5, 7) |  | 5(3, 6.5) | 6(4.3, 7) | .300 |  | 6(3, 6) | 6(4, 7) | .401 |  | 5.5(3.8, 6.3) | 6(3, 7) | .870 |
| Check visual field by confrontation test | 8(7.5, 9) |  | 9(7.5, 9) | 8(7.3, 9) | .306 |  | 9(7, 9) | 8(7.5, 9) | .639 |  | 8.5(6.8, 9) | 8(8, 9) | .961 |
| Check pupil size and shape | 9(8, 9) |  | 9(8, 9) | 9(8.3, 9) | .844 |  | 9(8, 9) | 9(7.8, 9) | .793 |  | 9(8.8, 9) | 9(8, 9) | .381 |
| Check direct light reflex | 9(9, 9) |  | 9(9, 9) | 9(8.3, 9) | .595 |  | 9(9, 9) | 9(7.8, 9) | .347 |  | 9(8.8, 9) | 9(9, 9) | .795 |
| Check indirect light reflex (II, III) and relative afferent pupillary defect | 8(7, 9) |  | 8(7, 9) | 7.5(6.3, 8) | .151 |  | 8(7, 9) | 7.5(5.8, 8) | .178 |  | 8(6.8, 9) | 8(7, 8) | .554 |
| Check accommodation reflex | 7(5.5, 7) |  | 7(5, 7) | 7(6, 7) | .780 |  | 7(5, 7) | 7(5.5, 7.3) | .440 |  | 7(4.5, 7) | 7(6, 7) | .735 |
| Check eye fundus using fundoscope | 4(3, 7) |  | 4(3, 7) | 4.5(3, 7) | .770 |  | 4(3, 7) | 5(3, 7.3) | .416 |  | 5.5(3, 7) | 4(3, 7) | .834 |
| Check upper eye lid for ptosis | 8(7, 9) |  | 8(7.5, 9) | 7.5(7, 8) | .117 |  | 8(7, 9) | 7.5(6.8, 8) | .081 |  | 8.5(6.8, 9) | 8(7, 9) | .431 |
| Check eye movements | 9(9, 9) |  | 9(9, 9) | 9(8, 9) | .209 |  | 9(9, 9) | 9(8, 9) | .090 |  | 9(9, 9) | 9(8, 9) | .297 |
| Check eye saccadic or pursuit movement | 7(5, 8.5) |  | 7(5, 9) | 6(5, 7.8) | .217 |  | 7(5, 9) | 6.5(5, 8) | .625 |  | 8(4.8, 9) | 7(5, 8) | .514 |
| Check eye convergent or divergent movement | 7(4.5, 8) |  | 7(5, 9) | 5(4, 7.8) | .192 |  | 7(4, 9) | 6(4.8, 8) | .709 |  | 7(4.5, 8.3) | 5(4, 8) | .709 |
| Check Bielschowsky head tilt test | 5(3, 7) |  | 5(3, 7) | 5(3.3, 6.8) | .770 |  | 4(3, 7) | 5.5(3.8, 7) | .779 |  | 6(3, 7.3) | 5(3, 7) | .499 |
| Check vertical gaze | 6(5, 9) |  | 7(5, 9) | 6(4.3, 7.5) | .190 |  | 7(5, 9) | 5.5(3.8, 6.5) | .072 |  | 6.5(3.5, 9) | 6(5, 9) | .833 |
| Check nystagmus | 8(6, 9) |  | 8(6.5, 9) | 7(6, 8.8) | .466 |  | 8(7, 9) | 7.5(5.8, 9) | .671 |  | 8(6.8, 9) | 7(6, 9) | .795 |
| Check cover and uncover test | 6(5, 8) |  | 6(5, 8.5) | 6(5, 7) | .622 |  | 6(5, 8) | 6.5(4.8, 7.3) | .710 |  | 6(2.8, 9) | 6(5, 7) | .908 |
| Check optokinetic nystagmus | 6(4.5, 8) |  | 6(4.5 ,8) | 6(4.3, 7.8) | .805 |  | 6(4, 8) | 6(4.5, 8) | .908 |  | 7(2.5, 8) | 6(5, 7) | .676 |
| Clenched teeth | 8(5, 8.5) |  | 7(4.5, 9) | 8(5.3, 8) | .821 |  | 7(5, 9) | 8(5, 8.3) | .797 |  | 8(5.3, 9) | 6(5, 8) | .574 |
| Check facial sensation by cotton swab on forehead/cheeks/jaws while eyes closed | 9(7, 9) |  | 9(6, 9) | 8.5(7.3, 9) | .961 |  | 9(6, 9) | 8(7, 9) | .545 |  | 8.5(6, 9) | 9(7, 9) | .512 |
| Check onion skin sensation | 7(5, 8.5) |  | 6(5, 9) | 7(6, 8) | .752 |  | 6(5, 9) | 7.5(5.8, 8.3) | .657 |  | 6(5, 9) | 7(5, 8) | .708 |
| Check jaw jerk | 7(5.5, 8.5) |  | 7(4.5, 8.5) | 7.5(7, 8.8) | .280 |  | 7(5, 9) | 7.5(7, 8.3) | .375 |  | 6.5(4.8, 8.3) | 7(6, 9) | .401 |
| Check cornea reflex with cotton wool | 8(6, 9) |  | 7(4, 9) | 8(6.3, 9) | .352 |  | 7(5, 9) | 8(6, 9) | .510 |  | 7.5(6.8, 9) | 8(5, 9) | .888 |
| Check facial nerve function by raising eyebrows/closing eyes tightly/smiling/ showing teeth | 9(8, 9) |  | 9(7.5, 9) | 9(8, 9) | .959 |  | 9(8, 9) | 8.5(8, 9) | .596 |  | 9(7.8, 9) | 9(8, 9) | .874 |
| Check taste | 5(2, 6) |  | 5(2, 6) | 4(2, 6) | .858 |  | 5(2, 6) | 5.5(2.8, 6.3) | .443 |  | 5(2, 6.3) | 5(2, 6) | .981 |
| Check lacrimation / salivation | 5(3, 7) |  | 5(2.5, 6) | 5.5(3.5, 7.8) | .107 |  | 4(3, 6) | 6.5(5, 8) | **.016** |  | 4.5(1.8, 6.3) | 5(3, 7) | .341 |
| Check hearing by Calibrated finger rub auditory screening test | 8(5.5, 9) |  | 8(5, 9) | 7.5(6.3, 8) | .928 |  | 8(5, 9) | 7.5(6.8, 8) | .981 |  | 7.5(4.3, 8.3) | 8(6, 9) | .482 |
| Check Weber /Rinne test by tuning fork | 7(5, 8) |  | 7(5, 8) | 7.5(5, 8) | .648 |  | 7(5, 8) | 8(5, 8.3) | .333 |  | 7(4.3, 8) | 8(5, 8) | .479 |
| Check vestibulo-ocular reflex (doll's eye test, head thrust) | 8(7, 9) |  | 8(7, 9) | 7.5(6.3, 9) | .550 |  | 8(7, 9) | 7.5(6, 9) | .445 |  | 8(6.5, 8.3) | 8(7, 9) | .340 |
| Check caloric test | 5(3, 6) |  | 4(1.5, 5.5) | 5(4, 6) | .122 |  | 4(2, 5) | 5.5(3.8, 6.3) | .099 |  | 4.5(2, 5.3) | 5(3, 6) | .500 |
| Check Hallpike's test | 5(3.5, 7) |  | 5(2, 7) | 5.5(4, 6.8) | .754 |  | 5(3, 7) | 6(3.8, 7) | .486 |  | 5.5(1, 6.3) | 5(4, 7) | .531 |
| Check ahh’ for uvula movement | 7(4, 8) |  | 7(4, 8.5) | 7(3.5, 8) | .788 |  | 7(6, 9) | 6(3, 8) | .416 |  | 7(5, 8.3) | 7(3, 8) | .834 |
| Touch pharyngeal wall with cotton wool stick (Gag reflex) | 8(6, 9) |  | 7(4.5, 9) | 9(7.3, 9) | **.036** |  | 8(5, 9) | 9(7, 9) | .162 |  | 7(5.8, 8.3) | 9(7, 9) | .135 |
| Check shrugging shoulders while pressing down on them or check head turning to each side against hand | 8(7, 9) |  | 8(5.5, 9) | 8(7, 9) | .448 |  | 8(6, 9) | 8(7, 9) | .520 |  | 7.5(5.5, 8.3) | 8(7, 9) | .182 |
| Check tongue movement | 8(8, 9) |  | 8(8, 9) | 8.5(8, 9) | .885 |  | 8(8, 9) | 8.5(7.8, 9) | 1.000 |  | 8(7.8, 9) | 9(8, 9) | .273 |
| Check the muscle strength distal and proximal on both sides | 9(8.5, 9) |  | 9(9, 9) | 9(8, 9) | .287 |  | 9(9, 9) | 9(7.5, 9) | .126 |  | 9(8.8, 9) | 9(8, 9) | .582 |
| Check the muscle strength of different myotomes | 8(6, 8.5) |  | 7(5, 8.5) | 8(7, 8.8) | .203 |  | 7(5, 9) | 8(6.8, 8.3) | .346 |  | 7.5(5.8, 9) | 8(6, 8) | .869 |
| Check the muscle strength of different nerves | 7(5.5, 9) |  | 6(5, 8.5) | 8(7, 9) | **.026** |  | 6(5, 9) | 8(6.8, 9) | .064 |  | 6(4.5, 9) | 8(6, 9) | .301 |
| Check muscle bulk and volume | 7(5, 8) |  | 7(5, 8.5) | 6.5(5.3, 7.8) | .530 |  | 7(5, 9) | 6.5(4.8, 7.3) | .307 |  | 7.5(3.8, 9) | 7(5, 8) | .710 |
| Check pronator drift | 8(6.5, 9) |  | 8(8, 9) | 7.5(6, 8.8) | .151 |  | 9(8, 9) | 7(5.8, 8) | .019 |  | 8.5(7.5, 9) | 8(6, 9) | .337 |
| Check Gower sign | 7(6.5, 9) |  | 8(6.5, 9) | 7(5.5, 8) | .116 |  | 8(7, 9) | 7(4.5, 8) | .053 |  | 8.5(6.8, 9) | 7(5, 8) | .103 |
| Could observe fasciculation | 7(5, 9) |  | 8(5, 9) | 7(6, 8) | .652 |  | 8(5, 9) | 7(5.8, 8) | .469 |  | 7.5(4.8, 9) | 7(6, 9) | .870 |
| Check light touchat arms/hands and legs/feet on both sides | 8(6, 9) |  | 7(5.5, 8.5) | 8(6.3, 9) | .139 |  | 7(6, 9) | 8(6, 9) | .239 |  | 7.5(6, 8.3) | 8(6, 9) | .814 |
| Check pinprick sensations, and compare the sensations between left/right side and proximal/distal side | 8(8, 9) |  | 9(8, 9) | 8(6.5, 9) | .223 |  | 9(8, 9) | 8(6, 9) | .151 |  | 8.5(7.8, 9) | 8(8, 9) | .785 |
| Check temperature sensations, and compare the sensations between left/right side and proximal/distal side | 7(5, 8) |  | 7(5, 8.5) | 6.5(5, 8) | .911 |  | 7(5, 9) | 6.5(5, 8) | .907 |  | 6.5(4.8, 8.3) | 7(5, 8) | .852 |
| Check vibration sensations using the tuning fork and compare the sensations between left/right side and proximal/distal side | 8(6, 9) |  | 8(6.5, 9) | 8(6,8. 8) | .927 |  | 8(6, 9) | 8(6, 8.3) | .887 |  | 8(6.8, 9) | 8(6, 9) | .814 |
| Check joint position sensation | 8(6.5, 9) |  | 9(6.5, 9) | 8(6.3, 8.8) | .441 |  | 9(7, 9) | 7.5(6, 8.3) | .246 |  | 9(6.8, 9) | 8(6, 9) | .483 |
| Check the truncal sensation of different dermatomes | 7(6, 9) |  | 7(5.5, 8.5) | 7.5(6.3, 9) | .350 |  | 7(6, 9) | 7.5(6, 9) | .539 |  | 7(5.3, 9) | 7(6, 9) | .654 |
| Check cortical sensation | 7(5, 8) |  | 7(5, 8) | 7(5.3, 8) | .787 |  | 7(5, 8) | 7(5.8, 8) | .889 |  | 7.5(5.8, 8.3) | 6(5, 8) | .327 |
| Check biceps, triceps, brachioradialis, patellar, and Achilles reflex | 9(9, 9) |  | 9(9, 9) | 9(9, 9) | .416 |  | 9(9, 9) | 9(9, 9) | .592 |  | 9(8.5, 9) | 9(9, 9) | .491 |
| Perform method of reinforcing the patellar reflex | 7(6, 9) |  | 7(6, 9) | 7(6, 8.8) | .633 |  | 7(6, 9) | 7(5.5, 8.3) | .540 |  | 8(7, 9) | 7(5, 9) | .220 |
| Check finger flexor | 6(5, 7.5) |  | 6(5, 8.5) | 6.5(4.3, 7) | .544 |  | 6(5, 8) | 7(3.8, 7) | .745 |  | 7(4.8, 8.3) | 6(5, 7) | .456 |
| Check pectoralis reflex | 5(4, 7) |  | 5(4, 7.5) | 6(4.3, 7) | .670 |  | 5(4, 7) | 6(4.8, 7) | .317 |  | 5.5(4, 8) | 5(4, 7) | .710 |
| Check Babinski sign | 9(9, 9) |  | 9(9, 9) | 9(8.3, 9) | .418 |  | 9(9, 9) | 9(8, 9) | .235 |  | 9(9, 9) | 9(9, 9) | .442 |
| Check Hoffmann' reflex | 8(7, 9) |  | 9(5.5, 9) | 8(7.3, 8.8) | .780 |  | 8(6, 9) | 8(8, 9) | .664 |  | 9(5.8, 9) | 8(7, 9) | .515 |
| Check clonus | 7(6, 9) |  | 8(6, 9) | 6.5(6, 7.8) | .248 |  | 7(6, 9) | 6.5(5.8, 8.3) | .398 |  | 8(6.5, 9) | 7(6, 8) | .166 |
| Check finger nose finger test | 9(9, 9) |  | 9(9, 9) | 9(8.3, 9) | .418 |  | 9(9, 9) | 9(8, 9) | .235 |  | 9(9, 9) | 9(9, 9) | .442 |
| Check heel-knee-shin test | 9(8, 9) |  | 9(7.5, 9) | 9(8, 9) | .412 |  | 9(8, 9) | 9(8, 9) | .791 |  | 9(7.8, 9) | 9(8, 9) | .690 |
| Check rapid alternative movement test | 9(7, 9) |  | 9(7.5, 9) | 8.5(7, 9) | .733 |  | 9(7, 9) | 9(7.8, 9) | .545 |  | 9(7.5, 9) | 9(7, 9) | .762 |
| Check muscle tones | 7(5.5, 9) |  | 7(5, 8.5) | 8(6, 9) | .364 |  | 7(5, 8) | 8.5(6.8, 9) | .091 |  | 8(5, 9) | 7(6, 9) | .724 |
| Check scanning speech | 7(5, 8) |  | 6(5, 8) | 7(5.3, 8) | .736 |  | 6(5, 8) | 7(5.5, 8) | .871 |  | 6.5(5, 8.3) | 7(5, 8) | .745 |
| Check rigidity or spasticity in upper/lower limbs and my neck | 8(5.5, 9) |  | 8(7.5, 9) | 6(5, 8.8) | .090 |  | 8(6, 9) | 6.5(5, 9) | .318 |  | 8(6.5, 9) | 8(5, 9) | .634 |
| Check bradykinesia by finger tapping movement | 7(5.5, 9) |  | 9(6.5, 9) | 6.5(5, 7.8) | .090 |  | 8(6, 9) | 7(4.8, 8.3) | .296 |  | 7.5(5.3, 9) | 7(5, 9) | .906 |
| Check resting tremor by counting number when eye closed | 6(5, 8.5) |  | 7(5, 9) | 6(5, 7.5) | .368 |  | 7(5, 9) | 6(4.8, 8.3) | .560 |  | 6.5(4, 9) | 6(5, 8) | .907 |
| Check pull test | 5(5, 8) |  | 6(5, 8) | 5(4.3, 7.8) | .229 |  | 6(5, 8) | 5(4, 8) | .384 |  | 6.5(4.8, 9) | 5(5, 8) | .359 |
| Describe the phenomenology of abnormal movements, including dystonia, spasticity, rigidity, tremor, chorea, ballism, and athetosis | 7(4.5, 8) |  | 7(4.5, 9) | 5.5(3.5, 7) | .139 |  | 7(4, 9) | 5.5(4.5, 7) | .265 |  | 7.5(4, 9) | 5(5, 7) | .185 |
| Check unified Parkinson's disease rating scale motor part | 5(3, 7) |  | 5(2, 7) | 5(3, 7) | .982 |  | 5(3 ,7) | 5.5(3, 7) | .608 |  | 7(1, 7.5) | 5(3, 7) | .376 |
| Observe the gait (arm swing, walk on heels, walk on toes, turn en bloc) | 8(7, 9) |  | 9(5, 9) | 8(7, 9) | .832 |  | 9(6, 9) | 8(7, 8.3) | .340 |  | 9(5.5, 9) | 8(7, 9) | .769 |
| Check tandem gait | 9(8, 9) |  | 9(8.5, 9) | 8.5(7.3, 9) | .229 |  | 9(9, 9) | 8(6.8, 9) | .073 |  | 9(8.8, 9) | 9(7, 9) | .193 |
| Check Romberg test | 9(8, 9) |  | 9(8, 9) | 8.5(7.3, 9) | .386 |  | 9(8, 9) | 8(7, 9) | .139 |  | 9(8.8, 9) | 9(7, 9) | .154 |
| Ask about urine or stool incontinence | 9(8, 9) |  | 9(8, 9) | 9(8.3, 9) | .778 |  | 9(8, 9) | 9(7.8, 9) | .861 |  | 9(7.8, 9) | 9(8, 9) | .861 |
| Check supine/standing blood pressure and heart rate | 8(5. 5,9) |  | 8(5, 9) | 8.5(7.3, 9) | .161 |  | 8(5, 9) | 8.5(6.8, 9) | .306 |  | 8(5, 9) | 8(6, 9) | .651 |
| Check meningeal irritation (Brudzinski's sign and Kernig's sign) | 9(8, 9) |  | 9(8.5, 9) | 9(7.3, 9) | .715 |  | 9(9, 9) | 9(6.5, 9) | .398 |  | 9(7.3, 9) | 9(8, 9) | .793 |
| Check National Institute of Health Stroke Scale | 7(5, 8) |  | 7(4.5, 8.5) | 6.5(5.3, 7.8) | .946 |  | 6(5, 8) | 7(5.8, 8.3) | .577 |  | 7(5.5, 8.3) | 6(5, 8) | .403 |

*One panelist did not complete the survey. Data were analyzed using Mann–Whitney U test. Bold p values are significant.
