# Supplementary material for: Core neurological examination items for neurology clerks: A modified Delphi study with a grass-roots approach
Source: PLoS One. 2018 May 17;13(5):e0197463. doi: 10.1371/journal.pone.0197463 (PMC5957356; doi:10.1371/journal.pone.0197463)
Supplement: S5 Table — (DOCX) [file pone.0197463.s005.docx]

S5 Table. Results of the second round modified Delphi process

|  | Overall |  | Tutors | learners |  |  | Neurologist | Non-neurologists |  |  | Medical center | Others |  |
| --- | --- | --- | --- | --- | --- | --- | --- | --- | --- | --- | --- | --- | --- |
|  | (n = 29*) |  | (n = 17) | (n = 12) | p |  | (n = 19) | (n = 10) | p |  | (n = 10) | (n = 19) | p |
| Items of NE | Median(Q1, Q3) |  | Median(Q1, Q3) | Median(Q1, Q3) | value |  | Median(Q1, Q3) | Median(Q1, Q3) | value |  | Median(Q1, Q3) | Median(Q1, Q3) | value |
| Check speech volume, pitch, rhythm | 4(3, 5) |  | 4(2.5, 5) | 4(3, 5.8) | .982 |  | 4(3, 5) | 4(2.8, 5.5) | .889 |  | 4(2.8, 5) | 4(3, 6) | .675 |
| Check glabellar sign  and palmomental reflexes | 5(4, 7) |  | 5(4, 7) | 4(3, 6.8) | .280 |  | 5(4, 7) | 4.5(3, 7) | .658 |  | 5.5(4, 7) | 4(3, 7) | .529 |
| Check smell by vinaigrette | 5(2.5, 6.5) |  | 5(2, 6.5) | 4.5(3, 6.8) | .823 |  | 5(2, 7) | 4.5(2.8, 6.3) | .799 |  | 4(2, 6) | 5(3, 7) | .366 |
| Check color vision | 5(3.5, 7) |  | 5(3, 7) | 6(5, 6.8) | .516 |  | 5(3, 7) | 5.5(4.5, 6.3) | .926 |  | 5(2.8, 6.3) | 6(5, 7) | .157 |
| Check eye fundus using fundoscope | 5(3.5, 7) |  | 5(3.5, 7.5) | 5(3.3, 6.8) | 1.000 |  | 5(3, 7) | 5(4.5, 7) | .387 |  | 4.5(3.5, 7.3) | 5(3, 7) | .743 |
| Check Bielschowsky head tilt test | 5(3.5, 6) |  | 5(4, 6.5) | 5(3, 6) | .445 |  | 5(4, 6) | 5(2.8, 6) | .926 |  | 4(3, 6.3) | 5(4, 6) | .328 |
| Check vertical gaze | 6(4.5, 8) |  | 7(4.5, 8.5) | 5(4.3, 7.5) | .209 |  | 7(5, 9) | 5(3.8, 6.5) | .094 |  | 6(4, 7.3) | 6(5, 8) | .471 |
| Check cover and uncover test | 6(4, 8) |  | 6(4.5, 8) | 5(3.3, 6.8) | .179 |  | 6(4, 8) | 5(2.8, 6.5) | .202 |  | 6(3.5, 7.3) | 6(4, 8) | .643 |
| Check optokinetic nystagmus | 6(4.5, 8) |  | 6(4, 8) | 6(5, 7.8) | .806 |  | 6(4, 8) | 6(5, 8) | .516 |  | 5.5(3, 7.3) | 6(5, 8) | .342 |
| Check taste | 4(3, 5.5) |  | 4(2.5, 5.5) | 4(3, 5.8) | .929 |  | 4(3, 6) | 3.5(2.8, 5.3) | .516 |  | 4(2, 5) | 4(3, 6) | .353 |
| Check lacrimation / salivation | 4(2, 5) |  | 3(1, 5) | 4.5(3.3, 5) | .065 |  | 3(1, 5) | 5(3, 5.3) | .069 |  | 2.5(1, 4.3) | 4(3, 5) | .062 |
| Check caloric test | 5(3, 6.5) |  | 4(1.5, 6.5) | 5(4.3, 6.5) | .226 |  | 4(2, 6) | 5(4.5, 7.3) | .193 |  | 3(1.8, 4.5) | 5(4, 7) | .051 |
| Check Hallpike's test | 5(3.5, 7) |  | 5(3, 7) | 5.5(4.3, 6.8) | .823 |  | 5(3, 7) | 5.5(3.8, 7) | .889 |  | 4(3, 7) | 6(5, 7) | .171 |
| Check finger flexor | 5(4, 7) |  | 6(4.5, 7) | 5(3.3, 7) | .458 |  | 5(5, 7) | 5.5(3, 7) | .624 |  | 5.5(3.8, 7) | 5(5, 7) | .592 |
| Check pectoralis reflex | 4(3, 5) |  | 3(2.5, 5) | 5(3, 5.8) | .198 |  | 4(3, 5) | 4.5(3, 6.3) | .387 |  | 3(2.8, 4.5) | 5(3, 5) | .168 |
| Check resting tremor by counting number when eye closed | 6(3.5, 7) |  | 6(3.5, 8) | 5.5(3.3, 6) | .561 |  | 6(4, 8) | 5.5(3, 6.3) | .562 |  | 5.5(2.8, 8.3) | 6(4,7) | .889 |
| Check pull test | 6(5, 7) |  | 6(5, 7.5) | 6(4.3, 7) | .653 |  | 6(5, 7) | 6(3.8, 7.3) | .544 |  | 6(4.8, 7) | 6(5, 8) | .816 |
| Check unified Parkinson's disease rating scale motor part | 6(3, 7) |  | 6(3.5, 7) | 5.5(3, 6.8) | .560 |  | 6(4, 7) | 5.5(2.8, 6.3) | .430 |  | 5.5(2.5, 7) | 6(3, 7) | .781 |
| Check the breathing sound | 8(6, 9) |  | 7(2.5, 9) | 9(7.5, 9) | **.010** |  | 7(3, 9) | 9(8.5, 9) | **.015** |  | 6(2, 7.3) | 9(7, 9) | **.004** |
| Check pulse and heart rate | 8(7, 9) |  | 8(5.5, 9) | 9(7.3, 9) | .257 |  | 8(6, 9) | 9(7, 9) | .352 |  | 7.5(4.5, 8.3) | 9(8, 9) | **.023** |
| Check Neck lymph node examination | 7(4, 9) |  | 6(2.5, 7.5) | 9(7, 9) | **.002** |  | 6(3, 8) | 9(7, 9) | **.005** |  | 4(2, 7.3) | 9(6, 9) | **.010** |
| Check Kayser-Fleischer rings | 7(5, 8) |  | 6(4.5, 8) | 7(6.3, 8) | .231 |  | 7(5, 8) | 7(6, 8) | .815 |  | 6.5(4.8, 8) | 7(6, 8) | .590 |
| Understanding the definition of coma, semi-coma, stupor, confusion, delirium, and dementia. | 8(8, 9) |  | 8(7.5, 9) | 9(8, 9) | .052 |  | 8(8, 9) | 9(8, 9) | .209 |  | 8(6.5, 8.3) | 9(8, 9) | **.016** |
| Clock drawing test | 7(6, 8) |  | 7(5.5, 8) | 6.5(6, 7) | .651 |  | 7(6, 8) | 6.5(5.8, 7) | .426 |  | 6.5(5, 8) | 7(6, 8) | .325 |
| Understanding the Horner syndrome | 8(7, 9) |  | 9(7, 9) | 8(7.3, 9) | .924 |  | 9(7, 9) | 8(7.8, 9) | .961 |  | 8(7, 9) | 9(7, 9) | .476 |
| Check superficial abdominal reflex | 5(3, 7.5) |  | 5(3, 7) | 6(5, 8.8) | .142 |  | 5(3, 7) | 6(4.5, 8.3) | .304 |  | 5(3, 6.5) | 5(3, 8) | .591 |
| Check drawing circle, spiral, and line | 5(3.5, 7.5) |  | 6(4, 6.5) | 5(3, 8.8) | .982 |  | 6(4, 7) | 5(2.8, 8.3) | .642 |  | 5(2.5, 6) | 6(4, 9) | .163 |
| Understanding abnormal gaits | 8(6.5, 9) |  | 8(7, 9) | 8(6, 8.8) | .767 |  | 7(6, 9) | 8(6.8, 9) | .524 |  | 7(6.3, 8.3) | 8(6, 9) | .321 |
| Ask erection function | 5(3, 7) |  | 5(2.5, 5.5) | 6(4.5, 8.5) | **.035** |  | 5(3, 6) | 6(3.8, 7.5) | .157 |  | 5(2.8, 6.3) | 6(3, 7) | .430 |
| Starch test | 3(2, 4.5) |  | 2(1, 3.5) | 3.5(3, 6.5) | **.015** |  | 2(1, 4) | 3.5(2.8, 7) | **.040** |  | 2.5(1, 3.3) | 3(2, 5) | .155 |
| Straight leg raising test | 8(7, 9) |  | 8(7, 9) | 7.5(6.3, 9) | .678 |  | 8(7, 9) | 7.5(5.3, 9) | .519 |  | 7.5(7, 8) | 8(7, 9) | .488 |
| Assess basic mood condition | 7(5.5, 9) |  | 7(5, 8) | 8.5(7, 9) | **.019** |  | 7(5, 8) | 8.5(6.8, 9) | **.044** |  | 5.5(4.3, 7) | 8(7, 9) | **.003** |

*One panelist did not complete the survey. Data were analyzed using Mann–Whitney U test. Bold p values are significant.
