# Supplementary material for: Core neurological examination items for neurology clerks: A modified Delphi study with a grass-roots approach
Source: PLoS One. 2018 May 17;13(5):e0197463. doi: 10.1371/journal.pone.0197463 (PMC5957356; doi:10.1371/journal.pone.0197463)
Supplement: S6 Table — (DOCX) [file pone.0197463.s006.docx]

S6 Table. Summary of the 22 items discarded during the Delphi process

1. Check speech volume, pitch, and rhythm
2. Check glabellar sign and palmomental reflexes
3. Check smell by vinaigrette
4. Check color vision
5. Check eye fundus using fundoscope
6. Check Bielschowsky head tilt test
7. Check vertical gaze
8. Check cover and uncover test
9. Check optokinetic nystagmus
10. Check taste
11. Check lacrimation / salivation
12. Check caloric test
13. Check Hallpike's test
14. Check finger flexor
15. Check pectoralis reflex
16. Check resting tremor by counting number when eye closed
17. Check pull test
18. Check unified Parkinson's disease rating scale motor part
19. Check superficial abdominal reflex
20. Check drawing circle, spiral, and line
21. Ask erection function
22. Starch test
