# Supplementary material for: Core neurological examination items for neurology clerks: A modified Delphi study with a grass-roots approach
Source: PLoS One. 2018 May 17;13(5):e0197463. doi: 10.1371/journal.pone.0197463 (PMC5957356; doi:10.1371/journal.pone.0197463)
Supplement: S7 Table — (DOCX) [file pone.0197463.s007.docx]

S7 Table. Results of the modified Delphi process from the tutors’ opinions

|  |  |  |  |  | Rating scores |  |  |
| --- | --- | --- | --- | --- | --- | --- | --- |
| Categories |  |  | 9 (Strong agreement) |  | 8 (Agreement) |  | 7 (Agreement) |
| Physical examinations |  |  |  |  | 1. Check pulse and heart rate |  | 1. Listen to the heart sounds  2. Listen to carotid bruits  3. Check breathing sounds |
|  |  |  |  |  |  |  |  |
|  |  |  |  |  |  |  |  |
| Conscious and cognitive functions |  |  | 1. Glasgow coma scale |  | 1. Check complete Mini-Mental State Examination  2. Check language function (reading, writing, repetition, comprehension, fluency, naming)  3. Understanding the definition of coma, semi-coma, stupor, confusion, delirium, and dementia |  | 1. Check glabellar sign and palmomental reflex  2. Check hemi-neglect  3. Clock drawing test |
|  |  |  |  |  |  |  |  |
|  |  |  |  |  |  |  |  |
| Cranial nerves |  |  | 1. Check visual field by confrontation test  2. Check pupil size and shape  3. Check direct light reflex  4. Check eye movements  5. Check facial sensations  6. Check facial nerve function |  | 1. Check indirect light reflex and relative afferent pupillary defect  2. Check upper eye lid for ptosis  3. Check nystagmus  4. Check hearing by finger rub screening test  5. Check vestibulo-ocular reflex  6. Check shrugging shoulders or head turning to each side against hand  7. Check tongue movement |  | 1. Check accommodation reflex  2. Check eye saccadic or pursuit movement  3. Check eye convergent or divergent movement  4. Check vertical gaze  5. Clenched teeth  6. Check jaw jerk  7. Check cornea reflex  8. Check Weber-Rinne test  9. Check uvula movement  10. Gag reflex |
|  |  |  |  |  |  |  |  |
|  |  |  |  |  |  |  |  |
|  |  |  |  |  |  |  |  |
|  |  |  |  |  |  |  |  |
|  |  |  |  |  |  |  |  |
|  |  |  |  |  |  |  |  |
|  |  |  |  |  |  |  |  |
|  |  |  |  |  |  |  |  |
| Motor system |  |  | 1. Check the distal and proximal muscle strength (MRC grading) |  | 1. Check pronator drift  2. Check Gower sign  3. Could observe fasciculation |  | 1. Check the muscle strength of different myotomes  2. Check muscle bulk and volume |
|  |  |  |  |  |  |  |  |
|  |  |  |  |  |  |  |  |
| Sensation |  |  | 1. Check pinprick sensations, and compare the sensations between left/right side and proximal/distal side  2. Check joint position sensation |  | 1. Check vibration sensations and compare the sensations between left/right side and proximal/distal side |  | 1. Check light touch at arms/hands and legs/feet on both sides  2. Check temperature sensations, and compare the sensations between left/right side and proximal/distal side  3. Check the truncal sensation of different dermatomes  4. Check cortical sensation |
|  |  |  |  |  |  |  |  |
| Reflexes |  |  | 1. Check biceps, triceps, brachioradialis, patellar, and Achilles reflexes  2. Check Babinski sign  3. Check Hoffmann' reflex |  | 1. Check clonus |  | 1. Perform methods of reinforcing the patellar reflex |
|  |  |  |  |  |  |  |  |
| Cerebellum |  |  | 1. Check finger nose finger test  2. Check heel-knee-shin test  3. Check rapid alternative movement test |  |  |  | 1. Check muscle tone |
|  |  |  |  |  |  |  |  |
|  |  |  |  |  |  |  |  |
| Extrapyramidal systems |  |  | 1. Check bradykinesia by finger tapping movement |  | 1. Check rigidity or spasticity in upper/lower limbs and neck |  | 1.Check resting tremor by counting numbers when eye closed  2. Describe the phenomenology of abnormal movements, including dystonia, spasticity, rigidity, tremor, chorea, ballism, and athetosis |
|  |  |  |  |  |  |  |  |
| Gait and stance |  |  | 1. Observe the gait (arm swing, walk on heels, walk on toes, and turn en bloc)  2. Check tandem gait  3. Check Romberg test |  | 1. Understanding abnormal gait, including hemiplegic gait, dystonic gait, scissors gait, wide base gait, festinating gait, gait apraxia |  |  |
|  |  |  |  |  |  | |  |
| Autonomic system |  |  | 1. Ask about urine or stool incontinence  2. Understanding the Horner syndrome |  | 1. Check supine/standing blood pressure and heart rate |  |  |
|  |  |  |  |  |  |  |  |
| Others |  |  | 1. Check meningeal irritation (Brudzinski's sign and Kernig's sign) |  | 1. Straight leg raising test |  | 1. Check National Institute of Health Stroke Scale  2. Assess basic mood condition |
|  |  |  |  |  |  |  |  |
|  |  |  |  |  |  |  |  |

MRC= [Medical Research Council](http://www.medicalcriteria.com/site/home/64-neurology/238-neuromrc.html)
